# The Enhanced Thermal Stability of (Mg_0.95_Ni_0.05_)_2_TiO_4_ Dielectric Ceramics Modified by a Multi-Phase Method

**DOI:** 10.3390/ma16082997

**Published:** 2023-04-10

**Authors:** Chun-Hsu Shen, Ting-Wei Shen, Tsai-Yu Hsieh, Kai-Chun Lan, Shen-Hsien Hsu, Ching-Hsuan Wang, Yu-Ting Lin, Wen-Fang Wu, Zong-Liang Tseng

**Affiliations:** 1 Department of Electronic Engineering, Ming Chuan University, Taoyuan City 333, Taiwan; 2 Department of Mechanical Engineering, National Taiwan University, Taipei 106, Taiwan; 3 Department of Electronic Engineering and Organic Electronics Research Center, Ming Chi University of Technology, New Taipei City 243, Taiwan

**Keywords:** thermal stability, radio frequency, dielectric ceramic

## Abstract

The thermal stability of (Mg_0.95_Ni_0.05_)_2_TiO_4_ dielectric ceramics has been improved by mixing with CaTiO_3_ phases owing to higher positive temperature coefficients. The pure (Mg_0.95_Ni_0.05_)_2_TiO_4_ and the mixture phase systems of CaTiO_3_-modified (Mg_0.95_Ni_0.05_)_2_TiO_4_ were verified by XRD diffraction patterns to ensure the crystallite of different phases. The microstructures of the CaTiO_3_-modified (Mg_0.95_Ni_0.05_)_2_TiO_4_ were observed by SEM and EDS to investigate the relation between element ratios and grains. As a result, it can be seen that the thermal stability of the CaTiO_3_-modified (Mg_0.95_Ni_0.05_)_2_TiO_4_ can be effectively enhanced, compared with the pure (Mg_0.95_Ni_0.05_)_2_TiO_4_. Moreover, the radio frequency dielectric performances of CaTiO_3_-modified (Mg_0.95_Ni_0.05_)_2_TiO_4_ dielectric ceramics are strongly dependent upon the density and the morphology of the specimens. The champion sample with the ratio of (Mg_0.95_Ni_0.05_)_2_TiO_4_ and CaTiO_3_ of 0.92:0.08 showed an ε_r_ value of 19.2, an *Qf* value of 108,200 GHz, and a τ*_f_* value of −4.8 ppm/°C, which may encourage (Mg_0.95_Ni_0.05_)_2_TiO_4_ ceramics to broaden the range of novel applications and match the requirements of 5G or next-generation communication systems.

## 1. Introduction

As a result of the continuous evolution of science and technology, millimeter-wave technology has been extensively developed and considered as a branch of 5G communication or next-generation mobile communication technology. Due to a large amount of data transmission, a multilayer board is required as the main circuit board design, leading to materials which should possess both low dielectric loss and conductor loss characteristics [1,2,3,4,5]. Dielectric materials, to date, are a good choice for creating high-frequency components that have a higher dielectric constant (ε_r_), which can contribute to the miniaturization of components; high-quality factors (*Qf* values) can improve the energy of stored electromagnetic waves and the temperature coefficient of the resonance frequency (τ*_f_*) approaching zero, which can enhance the thermal stability of the components. For example, when a filter possesses the three abovementioned characteristics, the filter results in effective downsizing, lower dielectric loss rates, greater filtering activity, and greater stability, unaffected by the external ambient temperature [6,7].

The ceramic systems based on MgTiO_3_ have always received considerable attention and applications in the literature. For example, dielectric passive components of MgTiO_3_ in a communication system have been employed to be a resonator, duplex, filter, and antenna [8,9,10,11,12]. Therefore, it is considerably important to enhance the dielectric performance of MgTiO_3_. When the ratio of Mg and Ti is 2:1, the binary titanate ceramic Mg_2_TiO_4_ possesses a spinel-type structure that belongs to the cubic phase with Fd-3m space group (2 2 7). It demonstrates an ε_r_ value of 14, a *Qf* value of 150,000 GHz, and a τ*_f_* value of −50 ppm/°C [13,14,15]. In addition, to further improve the dielectric characteristics, the method of partial substitution that selects Ni^2+^ (0.069 nm) with a radius resembling Mg^2+^ (0.078 nm) to perform the substitution was presented [16]. When the Mg^2+^ ions were replaced by Ni^2+^ ions to form (Mg, Ni)_2_TiO_4_ compositions, the (Mg_0.95_Ni_0.05_)_2_TiO_4_ compositions possessing a spinel-type structure presented a good dielectric performance with an ε_r_ value of 16.43, a *Qf* value of 238,000 GHz, and a τ*_f_* value of −55 ppm/°C [17]. Moreover, an inferior (Mg_0.95_Ni_0.05_)TiO_3_ phase also appeared during the synthesizing of (Mg_0.95_Ni_0.05_)_2_TiO_4_, which may be attributed to the effect of the thermal decomposition mechanism [18]. However, the slight (Mg_0.95_Ni_0.05_)TiO_3_-doped (Mg_0.95_Ni_0.05_)_2_TiO_4_ possess a comparable performance, with an ε_r_ value of 17.2, a *Qf* value of 180,000 GHz, and a τ*_f_* value of −45 ppm/°C [19], compared to those of the pure (Mg_0.95_Ni_0.05_)_2_TiO_4_ composition [17].

On the other hand, a multi-phase method has been employed to modify the dielectric characteristics. In Mg_2_TiO_4_–SrTiO_3_ systems [20], Mg_2_TiO_4_ and SrTiO_3_ have spinal cubic (lattice parameters: a = 8.439 Å, space group Fd3m) (ICDD-PDF#00-003-0858) and cubic perovskite (ICDD-PDF#01-084-0443), respectively. When x increased, the peak intensity of SrTiO_3_ increased and the lattice parameters of Mg_2_TiO_4_ remained unchanged, which demonstrates that the two-phase system was used to modify relative permittivity in the Mg_2_TiO_4_–SrTiO_3_ system. In (Mg_0.95_Zn_0.05_)_2_TiO_4_–SrTiO_3_ systems [21], the lattice parameters had slight influence and remained unchanged after the SrTiO_3_ was added into (Mg_0.95_Zn_0.05_)_2_TiO_4_, which confirms that the presence of a two-phase system could effectively promote densification in the (Mg_0.95_Zn_0.05_)_2_TiO_4_ matrix. In addition, CaTiO_3_- and SrTiO_3_-modified (Mg_0.95_Co_0.05_)_2_TiO_4_ ceramics [22,23] were presented using a multi-phase method for low–loss dielectric properties at microwave frequencies. Furthermore, thermal stability of dielectric ceramics is another important factor in practical applications. However, few studies have been conducted to discuss the thermal stability of (Mg_0.95_Ni_0.05_)_2_TiO_4_. For this purpose, we have made great efforts to enhance the thermal stability factor of (Mg_0.95_Ni_0.05_)_2_TiO_4_ compositions using a perovskite structured SrTiO_3_ additive to form a two-phase system in our previous report [24].

In this study, we demonstrate the microstructure and radio frequency dielectric characteristics of the CaTiO_3_-modified (Mg_0.95_Ni_0.05_)_2_TiO_4_ ceramic system for enhancing its thermal stability factor using the multi-phase method. We attempted to add CaTiO_3_ with a high positive temperature coefficient to form a three-phase system and to compensate the negative temperature coefficient of (Mg_0.95_Ni_0.05_)_2_TiO_4_. X-ray diffraction (XRD), scanning electron microscopy (SEM), and energy-dispersive X-ray spectrometer (EDS) analyses were also employed to study the microstructure grain boundary, and compositions of the ceramic system. The radio frequency dielectric performances of the CaTiO_3_-modified (Mg_0.95_Ni_0.05_)_2_TiO_4_ were quantified and examined by employing the formation of muti-phase coexistence.

## 2. Materials and Method

In this study, (Mg_0.95_Ni_0.05_)_2_TiO_4_ and CaTiO_3_ were produced by a mixed-oxide solid-state reaction using the following high-purity chemical powders: magnesium oxide (MgO), nickel oxide (NiO), calcium carbonate (CaCO_3_), and titanium dioxide (TiO_2_). Because MgO is hygroscopic in nature, it first removes the moisture remaining at 600 °C for 2 h. The mixture of the above oxides was mixed according to the stoichiometries of (Mg_0.95_Ni_0.05_)_2_TiO_4_ and CaTiO_3_. They were then ball ground in distilled water (ball grinding medium) for 24 h. All mixed powders were parched in a kiln and pre-phased (calcine) at 1100 °C for 4 h in a high-temperature furnace. Then, the pre-phased reagents were mixed again according to the chemical molar ratio of (1–x) (Mg_0.95_Ni_0.05_)_2_TiO_4_∙xCaTiO_3_, and ball ground into a fine powder for 24 h. Then, polyvinyl alcohol (PVA 500; Showa, Tokyo, Japan) as a binder was added to the calcined powder, uniformly granulated, screened with a 100-mesh screen, and compressed with a pressure of 200 MPa to form a tablet form with a height of 0.5 cm and diameter of 1.1 cm. The sintering temperatures of the tablets were set at 1300~1425 °C for 4 h in air. The risen and dropped temperature rates were set at 10 °C/min for all samples.

The crystallization-phase observation of the pre-phased powder and mixture compositions were analyzed by XRD (Rigaku D/Max III. V., Tx for USA) The lattice constant was calculated using the Rietveld method to fit the XRD patterns. SEM (Philips XL-40FEG, Eindhoven, The Netherlands) was employed to observe the surface morphologies of samples, and EDS was utilized to demonstrate the different phases and compositions. The apparent densities of the samples were measured using the Archimedes method. The ε_r_ and *Qf* values at radio frequencies were measured by the Hakki–Coleman dielectric resonator method [25,26,27].

The measurement was mainly composed of a vector network analyzer (HP8757D, Agilent Technologies, Taipei, Taiwan) and sweep oscillator connections (HP8350B, Agilent Technologies, Taipei, Taiwan). The thermal stability ($\tau_{f}$ values) was evaluated with a temperature range from 20 to 80 °C. The following Formula (1) was utilized to obtain the τ*_f_* value (ppm/°C):

(1) $$\tau_{f}=\frac{f_{2}-f_{1}}{f_{1}\left(T_{2}-T_{1}\right)}\;\;$$

where *f*_1_ and *f*_2_ represent the resonant frequencies at *T*_1_ and *T*_2_, respectively.

## 3. Results and Discussion

**Figure 1:**
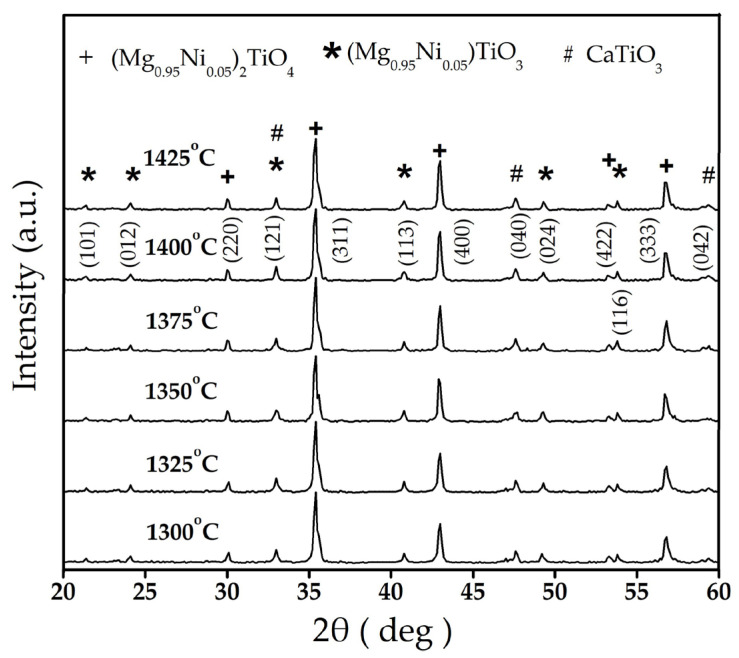
X-ray diffraction patterns of 0.92∙(Mg_0.95_Ni_0.05_)_2_TiO_4_∙0.08CaTiO_3_ sintered at different temperatures for 4 h.

Figure 1 presents the XRD patterns of the (1–x) (Mg_0.95_Ni_0.05_)_2_TiO_4_∙xCaTiO_3_ with an x value of 0.08 after sintering at different temperatures for 4 h. According to the JCPDS card, a three-phase system can be observed, which consisted of (Mg_0.95_Ni_0.05_)_2_TiO_4_ phase with a spinel-type structure as the primary crystalline phase (Mg_2_TiO_4_, ICDD–PDF#00-025-1157; lattice constants a = b = c = 0.84409 nm), (Mg_0.95_Ni_0.05_)TiO_3_ phase (MgTiO_3_, ICDD–PDF#00-006-0494), and a CaTiO_3_ phase with perovskite structure (JCPDS #22-0153). The lattice constant was slightly decreased with the increasing sintering temperature (a = b = c from 0.84005 to 0.83456 nm), indicating sharper main peaks of the (Mg_0.95_Ni_0.05_)_2_TiO_4_ phase and larger grain size. No other obvious phases and impurities were identified in Figure 1. In addition, the reason for the presence of the (Mg_0.95_Ni_0.05_)TiO_3_ phase is generally considered to be the uneven particle size of the initial materials, which increases the probability of secondary crystallization nucleation. Another reason for the formation of the (Mg_0.95_Ni_0.05_)TiO_3_ phase may be the effect of the thermal decomposition mechanism [18]. However, the ratio of (Mg_0.95_Ni_0.05_)TiO_3_ phase in the ceramics was decreased due to the increased grain boundary motion with the increasing sintering temperature, as shown in Table 1. Moreover, the (Mg_0.95_Ni_0.05_)TiO_3_ had no noticeable impact on the dielectric properties of (Mg_0.95_Ni_0.05_)_2_TiO_4_ in our previous study [19]. Therefore, the influence of the inferior (Mg_0.95_Ni_0.05_)TiO_3_ phase can be negligible when the sintering temperature is over 1350 °C.

**Figure 2:**
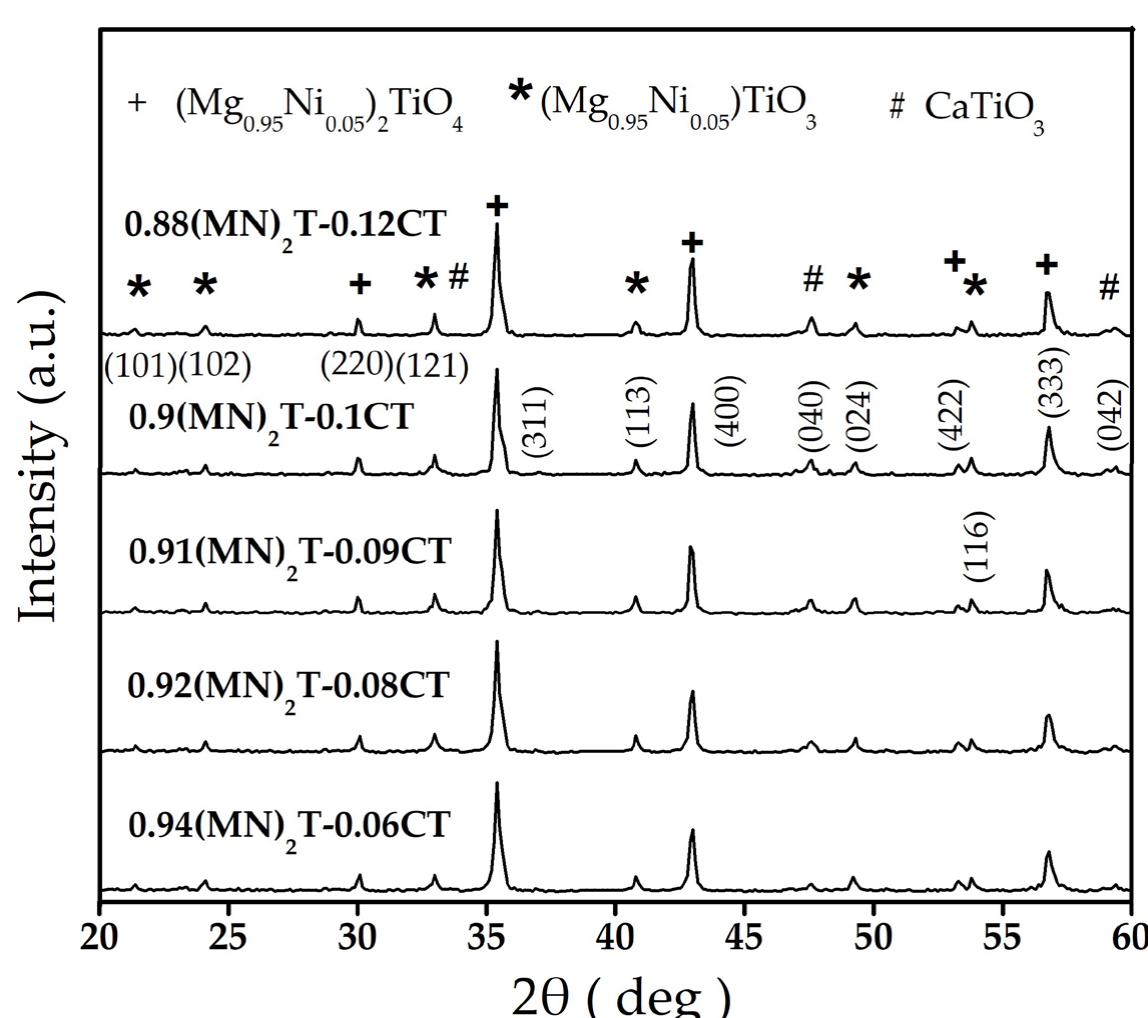
X-ray diffraction patterns of (1–x) (Mg_0.95_Ni_0.05_)_2_TiO_4_∙xCaTiO_3_ sintered at 1350 °C for 4 h.

Figure 2 presents the results of the XRD analysis of the (1–x) (Mg_0.95_Ni_0.05_)_2_TiO_4_ xCaTiO_3_ with different x values. It can be observed that the different contents have no obvious influence on the phase growth of the (Mg_0.95_Ni_0.05_)_2_TiO_4_. A few (Mg_0.95_Ni_0.05_)TiO_3_ peaks still existed in all patterns. As mentioned above, the effect of the inferior (Mg_0.95_Ni_0.05_)TiO_3_ phase on dielectric performance is quite slight and can be neglected. The lattice constant of the (Mg_0.95_Ni_0.05_)_2_TiO_4_ with a different amount of the CaTiO_3_ contents is presented in Table 2. It was also observed that all samples have a spinal cubic structure with the lattice constants (a = b = c) from 0.84005 to 0.83456 nm, indicating that the (Mg_0.95_Ni_0.05_)_2_TiO_4_ is still in the primary phase. When CaTiO_3_ was blended with (Mg_0.95_Ni_0.05_)_2_TiO_4_, no obvious influence on the lattice constants of (Mg_0.95_Ni_0.05_)_2_TiO_4_ could be found (Table 2). Furthermore, the growth of mixed phases in the one ceramic may cause a negative effect due to structural dissimilarities and the larger ionic radii values of Ca^2+^ (0.106 nm) compared to those of Mg^2+^ (0.078 nm) and Ni^2+^ (radii = 0.069 nm) [16]. However, the XRD analysis confirms the coexistence of the multiple phases without the structural dissimilarities in our samples.

**Figure 3:**
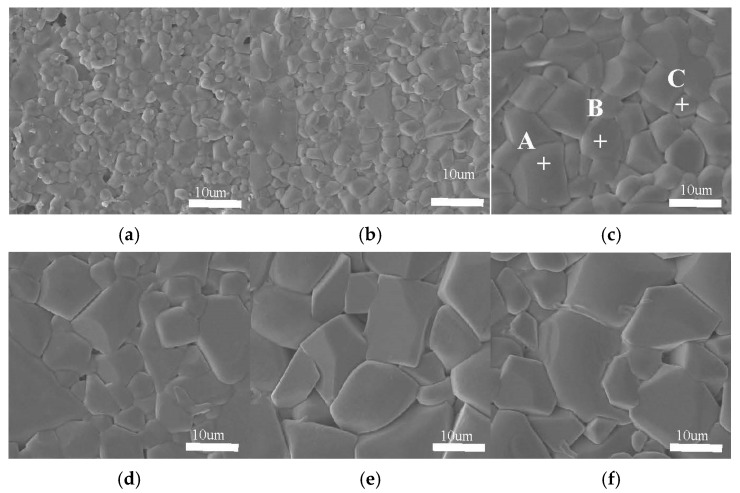
SEM images of 0.92∙(Mg_0.95_Ni_0.05_)_2_TiO_4_∙0.08CaTiO_3_ sintered at (**a**) 1300, (**b**) 1325, (**c**) 1350, (**d**) 1375, (**e**) 1400, and (**f**) 1425 °C for 4 h.

Figure 3 shows the SEM images of the morphologies of the 0.92∙(Mg_0.95_Ni_0.05_)_2_TiO_4_∙0.08CaTiO_3_ sintered at different temperatures. When the sintering temperature was increased, the grain size increased in Figure 3a–f, which is consistent with the XRD results (Figure 1). The pores reduced with the sintering temperature increasing from 1300 to 1350 °C. The reason for the increase in the grain size and the decrease in pores is the thermal drive energy, which enables connection and expands the neck between the grains. Therefore, Figure 3a–c exhibits the grain growth with better movement of the grain boundary and a more uniform and dense morphology. However, when the sintering temperature was increased from 1350 to 1425 °C, the grain size still increased, and the pore size became larger. This is attributed to the excessive extrusion between grains and air pressure under too-high sintering temperatures, which is similar with previous reports [20,21,22,23,24]. Furthermore, grains of 0.92∙(Mg_0.95_Ni_0.05_)_2_TiO_4_∙0.08CaTiO_3_ can be roughly divided into three shapes, as shown in Figure 3c. The EDS results of each grain are summarized in Table 3. Therefore, the different grains were identified as follows: spot A is (Mg_0.95_Ni_0.05_)TiO_3_; spot B is (Mg_0.95_Ni_0.05_)_2_TiO_4_’ and spot C is CaTiO_3_. The EDS results are consistent with the XRD analysis, verifying that the (1–x) (Mg_0.95_Ni_0.05_)_2_TiO_4_∙xCaTiO_3_ is a three-phase coexistence system.

**Figure 4:**
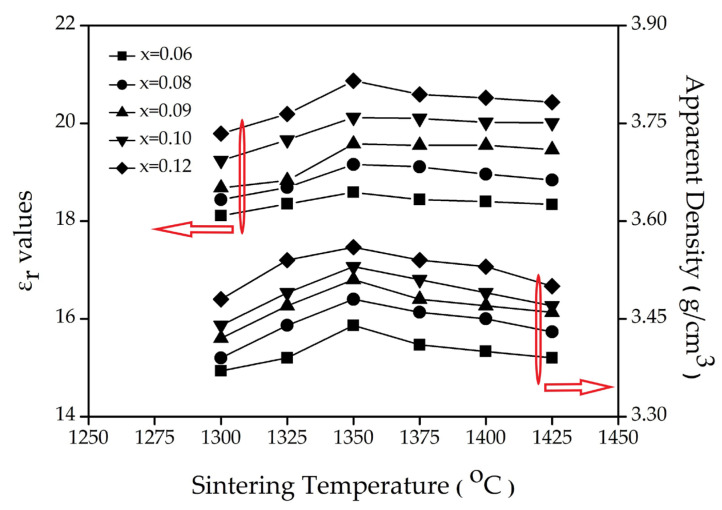
The apparent density and dielectric constant (ε*_r_* values) of the (1–x) (Mg_0.95_Ni_0.05_)_2_TiO_4_∙xCaTiO_3_ sintered at different temperatures.

Figure 4 shows the results of the apparent density and dielectric constant (ε*_r_* values) of the (1–x) (Mg_0.95_Ni_0.05_)_2_TiO_4_∙xCaTiO_3_ with different x values and temperatures. The apparent density was increased with the increasing x value because CaTiO_3_ (~4.036 g/cm^3^) possesses a higher density than that of (Mg_0.95_Ni_0.05_)_2_TiO_4_ (~3.49 g/cm^3^) [17]. Moreover, the apparent density value increased when the temperature increased from 1300 to 1350 °C, which is due to the grain growth (Figure 3a–c). However, the apparent density was reduced due to the enlarged pore size (Figure 3d–f) when the temperature increased from 1350 to 1425 °C. These results lead to the optimal sintering temperature of 1350 °C being obtained. Due to much higher dielectric constant (ε_r_) of (Mg_0.95_Ni_0.05_)_2_TiO_4_ (~16.4) and CaTiO_3_ (~170) than that of air (~1), the ε_r_ of the (1–x) (Mg_0.95_Ni_0.05_)_2_TiO_4_ xCaTiO_3_ correlates positively with apparent density. Therefore, the ε_r_ changed with the change in the apparent density and the x value. Therefore, the dielectric constant changes from 18.6 to 20.9 as x increases from 0.06 to 0.12, as shown in Table 4.

**Figure 5:**
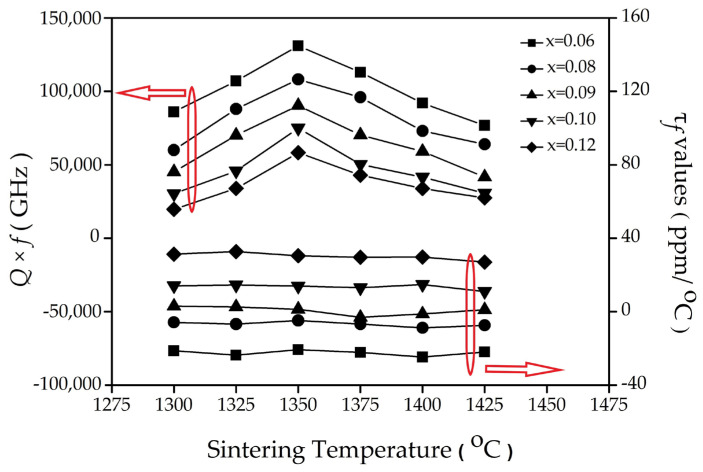
*Qf* and *τ_f_* values of the (1−x) (Mg_0.95_Ni_0.05_)_2_TiO_4_∙xCaTiO_3_ sintered at different temperatures for 4 h.

Figure 5 shows that the *Qf* value (quality factor) of the (1–x) (Mg_0.95_Ni_0.05_)_2_TiO_4_ xCaTiO_3_ decreased with the increasing the x value. It is attributed to the fact that the *Qf* value of CaTiO_3_ (~3600 GHz) was relatively smaller than that of (Mg_0.95_Ni_0.05_)_2_TiO_4_ (~238,000 GHz). In addition, the *Qf* of the (1–x) (Mg_0.95_Ni_0.05_)_2_TiO_4_∙xCaTiO_3_ rises at 1300–1350 °C and drops at 1350–1425 °C in Figure 5. Compared with Figure 4, the *Qf* of the (1–x) (Mg_0.95_Ni_0.05_)_2_TiO_4_ xCaTiO_3_ correlated positively with the ε_r_ and apparent density. Although there are many factors that impact the *Qf*, such as dielectric loss (which is caused by the second phase), oxygen vacancy, grain size, and porosity [28], the *Qf* is mainly dependent on the apparent density in our case. Similar with the results of the apparent density at different temperatures (Figure 4), the *Qf* also reached the optimal value at 1350 °C, as listed in Table 4. The *τ_f_* of the (1–x) (Mg_0.95_Ni_0.05_)_2_TiO_4_∙xCaTiO_3_ sintered at different temperatures seems to maintain a constant value, which is strongly dependent on the CaTiO_3_ contents (x value), as shown in Figure 5 and Table 4. The composition and phase determined the thermal stability of the (1–x) (Mg_0.95_Ni_0.05_)_2_TiO_4_∙xCaTiO_3_ because a high positive temperature coefficient of CaTiO_3_ can compensate the negative temperature coefficient of (Mg_0.95_Ni_0.05_)_2_TiO_4_. Therefore, the τ*_f_* was changed from −20.7 to 30.5 (ppm/°C) when x increased from 0.04 to 0.12, indicating that *τ_f_* = 0 is possible after controlling x. It is worth noting that the τ*_f_* was only −4.8 ppm/°C at x = 0.08 but the *Qf* was kept at 108,200 GHz.

## 4. Conclusions

In conclusion, the thermal stability of the CaTiO_3_-modified (Mg_0.95_Ni_0.05_)_2_TiO_4_ ceramic system was investigated. The dense morphology without pores can be obtained at 1350 °C, resulting in the optimal sintering temperature of the apparent density, ε*_r_*, and *Qf*. A high positive temperature coefficient of CaTiO_3_ can be used to improve the thermal stability of (Mg_0.95_Ni_0.05_)_2_TiO_4_. The τ*_f_* can be adjusted with changing the CaTiO_3_ contents, even closing to zero. The excellent dielectric characteristics of the 0.92∙(Mg_0.95_Ni_0.05_)_2_TiO_4_∙0.08CaTiO_3_ sintered at 1350 °C was presented with an ε_r_ of 19.2, an *Qf* of 108,200 GHz, and a τ*_f_* of −4.8 ppm/°C. Therefore, the CaTiO_3_-modified (Mg_0.95_Ni_0.05_)_2_TiO_4_ ceramic system showed high thermal stability and performance, suggesting the potential of these ceramics as dielectric substrate materials and radio frequency passive components in the microwave field to miniaturize components and transmit signals neglecting the temperature factor.

## Figures and Tables

**Table 1:** The lattice parameter and compositions of 0.92∙(Mg_0.95_Ni_0.05_)_2_TiO_4_ 0.08CaTiO_3_ sintered at different temperatures for 4 h. (Mg_0.95_Ni_0.05_)TiO_3_ and CaTiO_3_ ratios are determined by all peak areas of each phase.

Temperatures	a = b = c (nm)	(Mg_0.95_Ni_0.05_)TiO_3_ Ratio (%)	CaTiO_3_ Ratio (%)
1300	0.84005 ± 0.1033	17.4	11.6
1325	0.84005 ± 0.1033	17.2	13.4
1350	0.83986 ± 0.0972	17	13.5
1375	0.83456 ± 0.0995	14.9	13.8
1400	0.83456 ± 0.0995	14.7	13.8
1425	0.83456 ± 0.0995	14.4	14.1

**Table 2:** The lattice parameter and compositions of (1–x) (Mg_0.95_Ni_0.05_)_2_TiO_4_∙xCaTiO_3_ sintered at 1350 °C for 4 h. (Mg_0.95_Ni_0.05_)TiO_3_ and CaTiO_3_ ratios are determined by all peak areas of each phase.

x Values	a = b = c (nm)	(Mg_0.95_Ni_0.05_)TiO_4_ Ratio (%)	CaTiO_3_ Ratio (%)
0.06	0.84005 ± 0.1033	17.1	9.6
0.08	0.84005 ± 0.1033	17	13.5
0.09	0.83986 ± 0.0972	14.5	13.5
0.1	0.83456 ± 0.0995	13.3	13.7
0.12	0.83456 ± 0.0995	14	14.4

**Table 3:** Composition analysis from EDS results for spot A, B, and C in Figure 3c.

Atom (%)					
Spot	Mg	Ni	Ca	Ti	O
A	17.54	2.2	0	16.84	63.42
B	24.27	2.25	0	19.51	53.97
C	0	0	20.42	19.49	60.09

**Table 4:** Microwave dielectric performances of (1–x) (Mg_0.95_Ni_0.05_)_2_TiO_4_ xCaTiO_3_ at 1350 °C for 4 h.

x Values	Density (g/cm^3^)	ε_r_ Values	*Qf* Values (GHz)	*τ_f_* Values (ppm/°C)
0.06	3.44	18.6	131,000	−20.7
0.08	3.48	19.2	108,200	−4.8
0.09	3.51	19.6	90,000	1.3
0.1	3.53	20.1	75,000	13.9
0.12	3.56	20.9	58,000	30.5
